# Choroidal structural analysis in eyes with diabetic retinopathy and diabetic macular edema—A novel OCT based imaging biomarker

**DOI:** 10.1371/journal.pone.0207435

**Published:** 2018-12-11

**Authors:** Chanda Gupta, Roy Tan, Chitaranjan Mishra, Neha Khandelwal, Rajiv Raman, Ramasamy Kim, Rupesh Agrawal, Parveen Sen

**Affiliations:** 1 Medical Research Foundation, Sankara Nethralaya, Chennai, Tamil Nadu, India; 2 National Healthcare Group Eye Institute, Tan Tock Seng Hospital, Singapore; 3 Aravind Eye Hospital, Madurai, Tamil Nadu, India; 4 School of Material Science and Engineering, Nanyang Technological University, Singapore; Sung Kyun Kwan University School of Medicine at Samsung Medical Center Cancer Center, REPUBLIC OF KOREA

## Abstract

**Purpose:**

To evaluate structural changes in the choroid among patients with diabetic macular edema (DME), with varying grades of diabetic retinopathy (DR), using enhance depth imaging spectral domain optical coherence tomography (EDI SD-OCT) scans.

**Methods:**

A cross-sectional study was conducted on 82 eyes with DR and DME and 86 healthy control eyes. Eyes with DME were classified according to the severity of DR as per the international DR severity scale. Sub foveal choroidal thickness (SFCT)was obtained using EDI SD-OCT scans. These scans were binarized into luminal and stromal areas, to derive the choroidal vascularity index (CVI). CVI and SFCT were analyzed between the study and control group using paired-T test. Tukey’s test was used to correlate the differences in CVI and SFCT between different grades of DR. Further analysis was done to look for the effect of DR severity and type of DME on CVI as well as SFCT using correlation coefficient and linear regression analysis.

**Results:**

SFCT was significantly increased in eyes with DME as compared to the controls (334.47±51.81μm vs 284.53±56.45μm, p<0.001), and showed an ascending trend with worsening of DR, though this difference was not statistically significant [mild non-proliferative diabetic retinopathy (NPDR) = 304.33±40.39μm, moderate NPDR = 327.81±47.39μm, severe NPDR = 357.72±62.65μm, proliferative DR (PDR) = 334.59±47.4μm, p-0.09]. CVI was significantly decreased in DME with DR eyes as compared to controls (63.89±1.89 vs 67.51±2.86, p<0.001). CVI was also significantly decreased with worsening DR (mild NPDR = 66.38±0.3, moderate NPDR = 65.28±0.37, severe NPDR = 63.50±0.47, PDR = 61.27±0.9, p<0.001).

**Conclusion:**

SFCT and CVI are dynamic parameters that are affected by DME. Unlike CVI, SFCT is also affected by ocular and systemic factors like edema and hypertension. CVI may be a more accurate surrogate marker for DME and DR and can potentially be used to monitor the progression of DR.

## Introduction

Diabetic retinopathy (DR) and diabetic macular edema (DME) are the major causes of moderate visual impairment among the working age group characterized by abnormalities in retinal vessels and capillaries in diabetic patients [1]. It is well known that the choroid is an important vascular structure supplying the outer retinal layers, retinal pigment epithelium (RPE) and the photoreceptors [2]. Choroid plays a vital role in the pathogenesis of DR and DME. With the recent advent of newer optical coherence tomography (OCT) techniques such as enhanced depth imaging (EDI) and the swept source OCT (SS-OCT), high resolution scans of the retina and choroid can now be obtained in a non**-**invasive manner. EDI SD-OCT produces higher resolution images with increased depth of penetration, while SS-OCT uses longer wavelength and faster scanning speed, which allows for deep range imaging.

Several studies have attempted to compare the subfoveal choroidal thickness (SFCT) between patients with DR against normal patients [3–12]. However, there is no consensus in the results from these studies. A review article that looked at the current available literature on diabetic choroidopathy [13] concluded that it is unclear if choroidal changes in patients with diabetes are predictive, modulatory, causative or independent factors for DR, and there is inconclusive result from the clinical studies.

We believe that these inconsistent results are due to the fact that the choroidal thickness is a gross parameter and can be affected by many variables. There are several studies that have shown histopathological changes in the choroid in patients with DR [14–16]. Nagaoka et al analyzed the blood flow in the choroid using laser Doppler flowmetry and showed a decrease in choroidal blood flow in patients with non-proliferative diabetic retinopathy (NPDR), and even more so in patients with NPDR and DME [17]. In our study, we looked at this choroidal vasculature in more detail, analyzing the structural changes in the choroid as a whole as well its vascular density through an easily accessible and non-invasive approach. The vascularity of the choroid was derived by our previously validated tool of choroidal vascularity index (CVI) [18]. CVI has been shown to be more robust in studying changes in the choroid as compared to choroidal thickness [19]. CVI has been used to analyze the choroid in a cohort of diabetic patients from Singapore [20] and Korea [21]. In both the studies, patients with diabetes mellitus (DM) showed a decrease in CVI compared to healthy controls. There are limited studies published in the literature about the role of the choroid and application of CVI in eyes with DME with DR.

Our current study is the first ever comprehensive approach to analyze the structural changes in the choroid using SFCT and CVI in patients with DME with DR and correlate with ocular and systemic factors. In this study, with a large cohort of eyes from the South Indian population, we subdivided the study population of DME eyes into subgroups with varying severity of DR, classified DME into subtypes and performed comparative subgroup analyses.

## Materials and methods

### Study population

We conducted a cross sectional study of 82 eyes of 52 patients with treatment naïve DME with varying grades of DR and 86 eyes of 43 healthy control patients between 1^st^ September 2015 to 31^st^ December 2016. The patients with DME were recruited from a tertiary eye care hospital in Southern India. Healthy controls were recruited from the clinics with non-retina related issues from an another center in South India. Written informed consents for diagnostic and therapeutic procedures were obtained from all the patients and prior approval was obtained from the Institutional Ethical Committee Board of Medical Research Foundation and Aravind Medical Research Foundation. The study adhered to the tenets of Helsinki Declaration.

#### Sample size calculation

With a level of significance of 5% and the power of 80%, using the CVI values [19] for control group as 67.2 ± 0.16, for diabetes group as 65.1± 0.16 and the effect size of 2%, the minimum sample size was found to be 82 in each group. We included treatment naive DR patients with DME, diagnosed with mild to severe non-proliferative DR (NPDR) or proliferative DR (PDR). Patients with other concurrent retinal pathologies (age related macular degeneration, arterial or vein occlusions, uveitis), significant media haze, epiretinal membrane, tractional components and those who underwent intraocular surgery within last 6 months, were excluded from this study. All patients underwent a targeted ocular and systemic history taking, a detailed ophthalmic evaluation in the form of best corrected visual acuity (BCVA), slit lamp examination, intraocular pressure measurement with Goldmann applanation tonometry, a detailed indirect ophthalmoscopy, a 78 slit lamp biomicroscopic examination, fundus photography and fundus fluorescein angiography (FFA). Patients were diagnosed to have clinically significant DME according to ETDRS criteria [22] and further classification was done by SD-OCT. DR was classified into mild, moderate, severe NPDR or PDR according to the International DR disease severity scale [23]. Mean Ocular Perfusion Pressure (MOPP) was computed using intraocular pressure (IOP) and the systolic and diastolic blood pressure (SBP and DBP) readings. It was calculated as 2/3^rd^ of the difference between mean arterial pressure (MAP) and the baseline IOP.

### OCT image acquisition parameters assessments

Imaging of the choroid was performed using Spectralis HRA+OCT imaging device (Heidelberg Engineering, Germany). The SD-OCT imaging protocol comprised of 49 horizontal 9mm raster B-scans centered at the fovea per volume scan of 30° × 30°.

For each patient, EDI horizontal scan image passing through the fovea was obtained. The foveal center was defined by a hyper-reflective dot echo at the innermost retinal layer. The Spectralis software generates retinal thickness and volume maps with ETDRS grid (1mm, 3mm and 6mm) that gives retinal thickness and volume in the centre and in all the quadrants. Central macular thickness (CMT) was recorded from the retinal thickness ETDRS grid (centre 1 mm). Central foveal thickness (CFT) was manually measured using calliper tool as the distance between the internal limiting membrane and the anterior surface of the retinal pigment epithelium (RPE). Central macular volume (CMV) and Total macular volume (TMV) were recorded from the retinal volume map with the ETDRS grid (central 1mm and total 6mm respectively). Sub-foveal choroidal thickness (SFCT) was recorded as the vertical distance measured manually at the fovea using the caliper tool in the software, from the hyper reflective line of Bruch’s membrane to the hyper-reflective line of the chorio**-**scleral interface.

### DME classification

DME was classified into 3 patterns based on characteristics on SD-OCT [24] as diffuse retinal thickening showing increased retinal thickness with reduced intra-retinal reflectivity and expanded areas of lower reflectivity, cystoid macular edema (CME) showing intra retinal cystoid spaces at the macula and serous retinal detachment (SRD) showing sub-retinal fluid accumulation with distinct outer border of the detached retina. Images with more than one pattern of DME were categorised on the basis of predominant pattern of DME present.

### Image binarization

**Fig 1:**
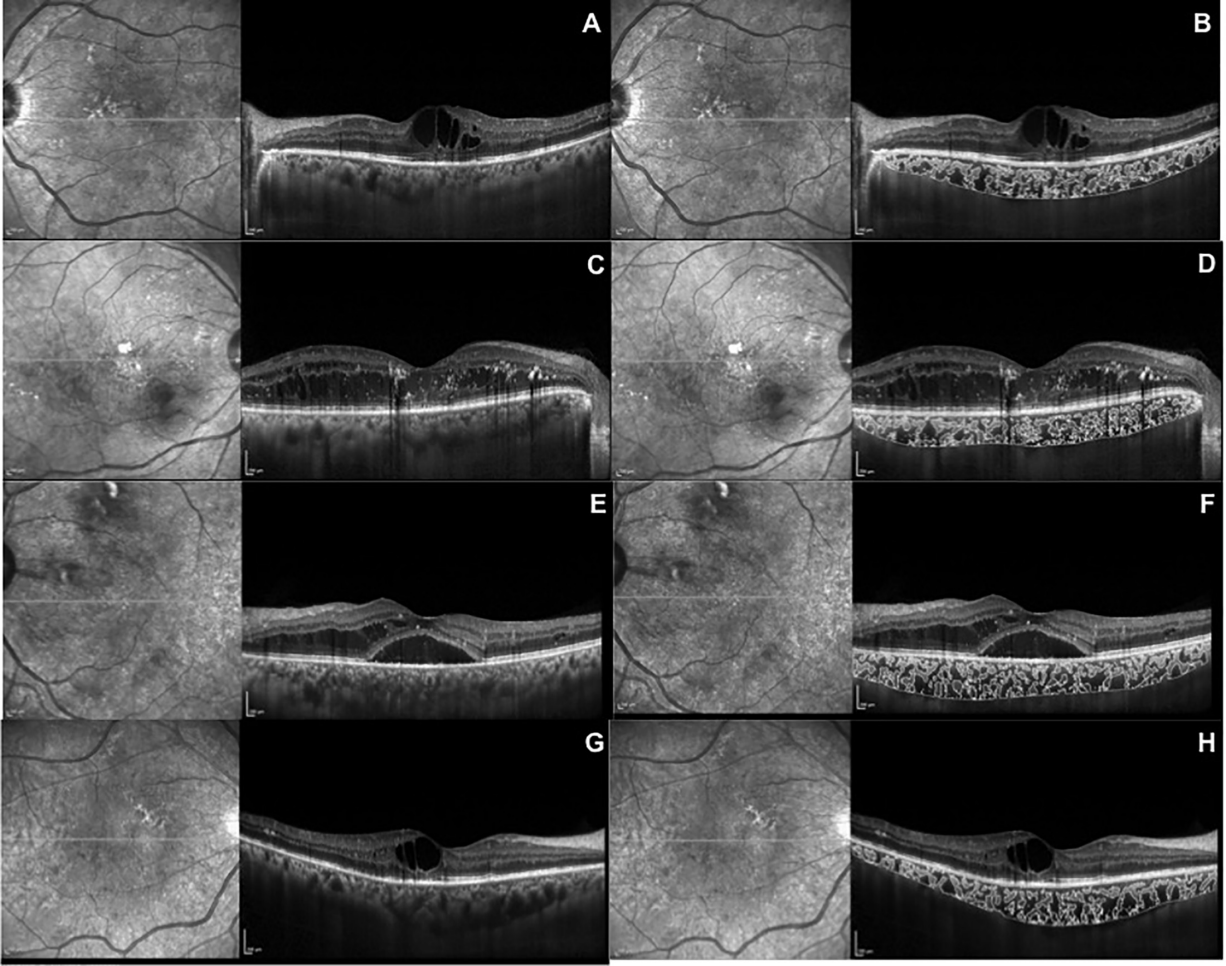
Image binarization of EDI OCT scans. **A-** Non segmented scan of patient with mild non proliferative diabetic retinopathy (NPDR); **B-** Segmented scan of patient with mild non NPDR**. C-** Non segmented scan of patient with moderate NPDR; **D-** segmented scan of patient with moderate NPDR**; E-** Non segmented scan of patient with severe NPDR; **F-** segmented scan of patient with severe NPDR; **G-** Non segmented scan of patient with proliferative diabetic retinopathy (PDR); **H-** Segmented scan of patient with PDR.

The entire length of the horizontal OCT scan was used for binarization, using the same protocol that was previously described by our group [18]. A trained grader (NK), who was masked to the patients’ information, performed the segmentation of the images. The OCT images obtained were uploaded and processed on a public domain software, Fiji Image J (version 1.47; http://imageJ.nih.gov/ij/). Total choroidal area (TCA) was defined as the area bounded by the RPE anteriorly and the chorio**-**scleral interface posteriorly, across the entire length of the scan. This area (TCA) was then added to the regions of interest (ROI) manager. Conversion of the images into 8 bit was then performed, followed by the application of Niblack auto local thresholding, to determine the mean pixel value for all the points [19]. Using a color threshold tool, we obtained dark pixels that represented the luminal area (LA), and light pixels that represented the stromal or interstitial area (SA) (Fig 1A, 1B, 1C, 1D, 1E, 1F, 1G and 1H).

### CVI assessment

CVI was then calculated using the ratio of LA/TCA. Images that did not have distinct chorio-scleral junction were excluded from the study. The SD-OCT images were analysed and compared for pattern of DME, CMT, CFT, CMV, TMV, SFCT and CVI.

### Statistical analysis

All analyses were performed in SPSS ver 20.0 (IBM corp.) and statistical significance was evaluated at 5% level. All quantitative variables were estimated using mean and standard deviation (SD). Qualitative or categorical variables were described as frequencies (n) and percentages (%). The BCVAs in all the eyes were converted to the logarithm of the minimal angle of resolution (logMAR) equivalent for analysis. SFCT and CVI were analyzed independently using linear mixed effects model, treating age and gender as covariates. A two-eyes design was followed with an aim to partition the variance of SFCT and CVI contributed by patients and that resulting from differences in eyes. Paired t-test and pearson’s correlation coefficient test were used for continuous and categorical variables respectively. Statistical significance of difference of estimated marginal means of SFCT and CVI between the two groups was obtained after adjusting for covariates. Tukey’s test was used to correlate the differences in CVI and SFCT between different grades of DR. In addition, correlation of CVI and SFCT was independently performed with BCVA and other parameters using scatter plots, obtaining the correlation coefficient and linear regression analysis.

## Results

Eighty-two eyes of DR patients with DME (study group) were compared with 86 eyes of patients without DR or DME (control group). The mean age of patients in the study group and control group were 55.8±6.8 years and 55.0±5.3 years respectively (p value = 0.40). In the study group, there were 43 males (52.4%) and female (47.6%), while in the control group, there were 46 males (53.5%) and 40 females (46.5%), with a p-value of 0.76 on Pearson’s chi-squared test (Table 1).

**Table 1:** Demographic characteristics of subjects in normal and DR with DME groups.

Characteristics	Groups		Statistic	P-value
	Normal (n = 86)	DR with DME (n = 82)		
**Age in years [N (%)]**				
***≤50 years***	20 (23.26)	16 (19.51)	0.16	0.69^a^
***>50 years***	66 (76.74)	66 (80.49)		
**Mean±SD**	54.98±5.32	55.77±6.82	-0.84	0.40^b^
**Gender [N (%)]**				
***Male***	46 (53.50)	43 (52.44)	1.46	0.76^a^
***Female***	40 (46.50)	39 (47.56)		
**Choroidal Vascularity Index (CVI) [Mean±SD] (%)**	67.51±2.86	63.89±1.89	9.73	**<0.001**^b^
**Subfoveal Choroidal Thickness (SFCT) [Mean±SD] (μm)**	284.53±56.4	334.48**±**51.82	-	**<0.001**^b^

^a^Obtained using Pearson’s chi-square test;

^b^Obtained using independent sample t-test. DME—Diabetic macular edema, DR- Diabetic retinopathy

The mean CVI and SFCT were analyzed and compared, among varying severity of DR, pattern of DME, presence of SRD, retinal thickness and volume, BCVA, mean ocular perfusion pressure (MOPP) and presence of hypertension. The mean SFCT in the control group was 284.5±56.5μm (163–471μm) and study group was 334.5±51.8μm (206–478μm) (p<0.001). The mean CVI in the study group (63.9 ± 1.9) was significantly lower than that in the control group (67.5 ± 2.9) (p <0.001). CVI decreased with increasing severity of DR as shown in Table 2.

**Table 2:** Descriptive statistics for CVI according to diabetic retinopathy grades.

Grade of DR	N (%)	CVI [Mean±SD]	SFCT [Mean±SD] (μm)
**No DR (controls)**	86 (51.19)	67.51±2.86*	284.53±56.45
**Mild NPDR**	6 (3.57)	66.38±0.31*	304.33±40.39
**Moderate NPDR**	36 (21.43)	65.28±0.37*	327.81±47.39
**Severe NPDR**	18 (10.17)	63.50±0.47*	357.72±62.65
**PDR**	22 (13.10)	61.27±0.96*	334.59±47.4
**Total**	168 (100)	-	-

*P-value: < 0.001; for CVI; P-value: 0.09 for SFCT obtained using one-way ANOVA; Similar superscripts indicate statistically significant difference of CVI; DR—diabetic retinopathy; PDR—Proliferative diabetic retinopathy

**Fig 2:**
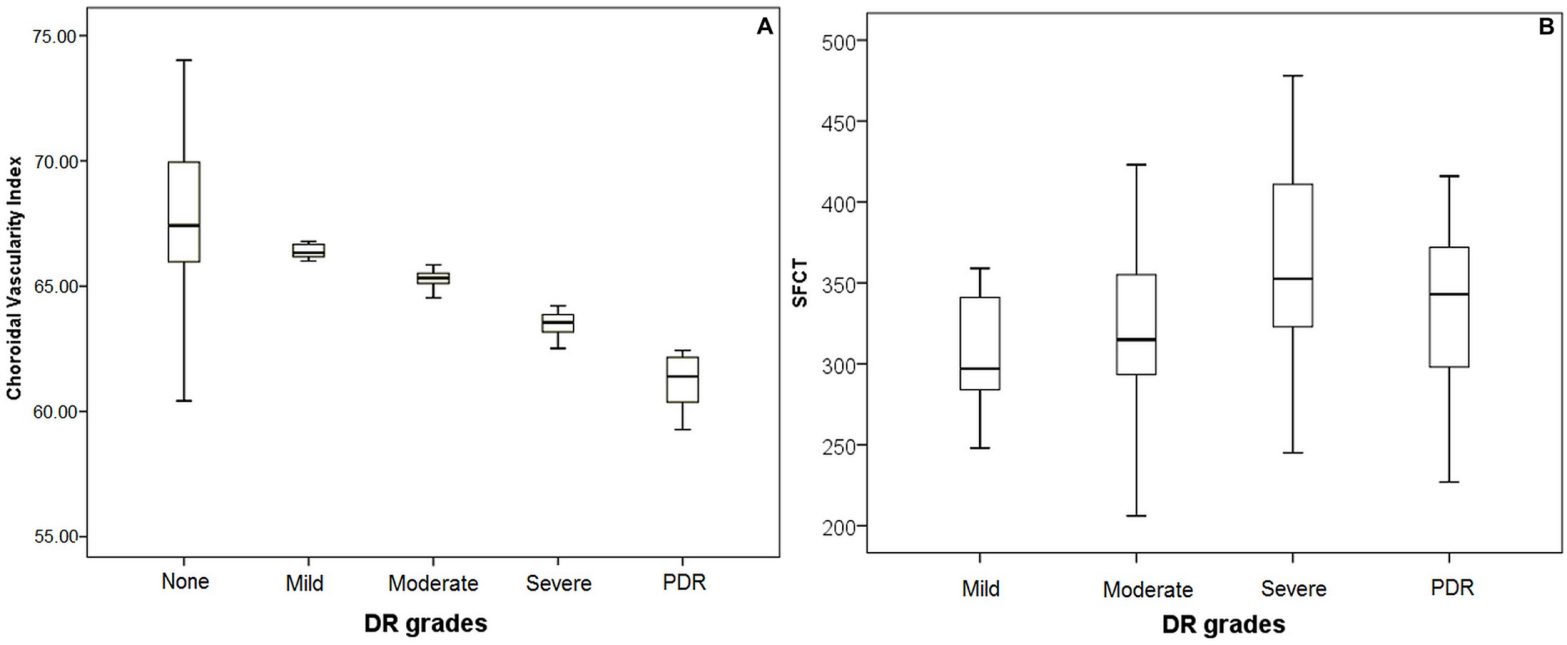
Correlation between grades of diabetic retinopathy (DR) and subfoveal choroidal thickness (SFCT) and choroidal vascularity index (CVI); A: Box-plot for CVI according to grades of DR; B: Box-plot for SFCT according to grades of DR.

Among the patients with DR, CVI was highest in eyes with mild DR (66.4±0.31), and lowest in eyes with PDR (61.3±0.96). There was statistically significant difference in the mean CVI across the DR severities and control group (p < 0.001). Pair wise comparison using Tukey’s test revealed that the mean CVI for moderate NPDR, severe NPDR and PDR groups differed significantly from that of mild NPDR and normal groups. SFCT was found to be increasing with severity of DR, though this difference was not found significant (Table 2). The correlation between grades of DR with CVI and SFCT is shown in Fig 2A and 2B.

Amongst the patterns of DME, SFCT was found to be significantly increased in cases with SRD (diffuse retinal thickening = 322.3±40.2μm, CME = 328.0±52.6μm and SRD = 377.7±56.7μm, p = 0.001) (Table 3).

**Table 3:** Descriptive statistics for SFCT and CVI according to pattern of DME.

Pattern of DME	N (%)	SFCT [Mean±SD] (μm)	CVI [Mean±SD] (%)
**Diffuse retinal thickening**	38 (46.34)	322.3±40.2*	63.7±2.0
**Cystoid macular edema (CME)**	29 (35.36)	328.0±52.6*	64.1±1.8
**Serous retinal detachment (SRD)**	15 (18.30)	377.7±56.7*	63.8±1.6

*P-value: < 0.001 for SFCT; P-value = 0.06 for CVI; obtained using one-way ANOVA.

Similar superscripts indicate statistically significant difference of CVI

**Fig 3:**
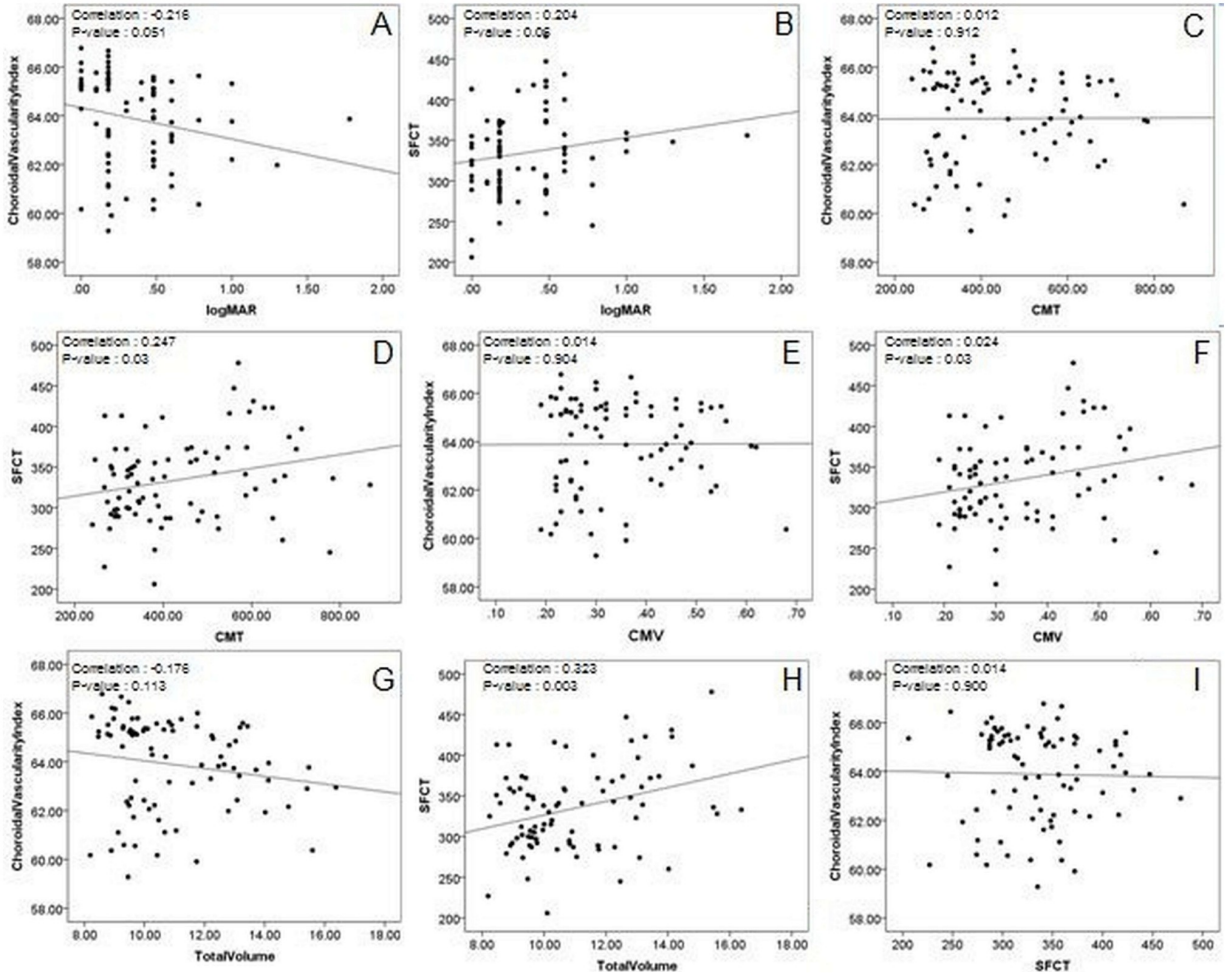
Scatter plots to demonstrate correlation between choroidal vascularity index (CVI) and subfoveal choroidal thickness (SFCT) with other variables including visual acuity (VA). **3A**: Scatter plot showing relationship between vision (logMAR VA) and CVI. **3B**: Scatter plot showing relationship between vision (logMAR VA) and SFCT**. 3C**: Scatter plot showing relationship between CMT and CVI. **3D**: Scatter plot showing relationship between CMT and SFCT**. 3E**: Scatter plot showing relationship between CMV and CVI. **3F**: Scatter plot showing relationship between CMV and SFCT. **3G**: Scatter plot showing relationship between Total volume (TMV) and CVI. **3H**: Scatter plot showing relationship between Total volume (TMV) and SFCT. **3I**: Scatter plot showing relationship between SFCT and CVI.

No significant relationship was found between patterns of DME and CVI (diffuse retinal thickening = 63.7±2.0, CME = 64.1±1.8 and SRD = 63.8±1.6, p = 0.60). The relationship between various clinical parameters and mean CVI, are shown through scatter plots (Fig 3A–3I).

The correlation coefficient (*r)* for VA and CVI was—0.21, which although was statistically insignificant (p = 0.05), it reflected a worsening BCVA with a decrease in CVI (Fig 3A). The correlation coefficient (r) for VA and SFCT was 0.20, which although was also statistically insignificant (p = 0.06), it reflected worsening BCVA with an increase in SFCT (Fig 3B). Similar results were obtained on both univariate and multivariate regression analysis. (Tables 4 and 5).

**Table 4:** Linear regression analyses of ocular and systemic factors associated with sub foveal choroidal thickness (SFCT).

	Univariate			Multivariate		
	Unstandardized β	Standardized β	P value	Unstandardized β	Standardized β	P value
**Ocular factors**						
**MOPP(mmHg)**	- 0.194	-0.026	0.814	-	-	-
**CVI** **logMAR VA**	-0.639 37.60	-0.023 0.204	0.836 0.065	- -	- -	- -
**CMT** **CMV**^a^ **TMV** **CFT**	0.085 105.47 8.47 0.050	0.247 0.240 0.323 0.167	**0.026** **0.030** **0.003** 0.135	0.182 - 4.645 -	0.529 - 0.177 -	0.263 - 0.449 -
**Systemic factors**						
**Age (year)**	0.670	0.070	0.380	0.684	0.071	0.368
**Gender:Male**	8.739	0.071	0.368	5.912	0.048	0.550
**DR with DME**^b^	49.94	0.421	**<0.001**	-	-	-
**Hypertension**	16.752	0.128	**0.001**	15.442	0.118	0.001

Age and gender were inputed in the multivariate analysis though they were insignificant on univariate analysis.

^a^CMV showed multicollinearity with CFT, and hence ignored from multivariate model.

^b^DME showed collinearity with HTN and hence was ignored from the model.

MOPP = Mean ocular perfusion pressure; CVI = Choroidal vascularity index; VA = Visual acuity;

CMT = Central macular thickness; CMV = Central macular volume; TMV = Total macular volume; CFT = Central foveal thickness; DME = Diabetic macular edema

**Table 5:** Linear regression analyses of ocular and systemic factors associated with choroidal vascularity index (CVI).

	Univariate			Multivariate		
	Unstandardized β	Standardized β	P value	Unstandardized β	Standardized β	P value
**Ocular factors**						
**MOPP (mmHg)**	0.030	0.111	0.323	0.053	-	-
**SFCT** **LogMAR VA**	-0.001 -1.140	-0.023 -0.170	0.836 0.126	0.005 -1.154	0.137 -	0.228 -
**CMT** **CMV**^a^ **TV** **CFT**	0.001 0.094 -0.159 0.001	0.004 0.006 -0.166 0.063	0.971 0.958 0.136 0.572	-0.001 - -0.294 0.003	- - - -	- - - -
**Systemic factors**						
**Age (year)**	0.005	0.011	0.892	0.006	0.012	0.865
**Gender:Male**	-1.443	-0.233	0.095	-0.989	-0.160	0.080
**DR with DME**^b^	-3.616	-0.599	<0.001	-	-	-
**Hypertension**	-2.547	-0.376	0.256	-	-	-

Age, gender and SFCT were inputed in the multivariate analysis though they were insignificant on univariate analysis.

^a^CMV showed collinearity with CFT, and hence ignored from multivariate model.

^b^DR showed collinearity with HTN and hence was ignored from the model.

MOPP = Mean ocular perfusion pressure; CVI = Choroidal vascularity index; SFCT = Subfoveal choroidal thickness;

VA = Visual acuity; CMT = Central macular thickness; CMV = Central macular volume; TMV = Total macular volume; CFT = Central foveal thickness; DME = Diabetic macular edema

A significant positive correlation was found between SFCT and OCT parameters such as CMT (p = 0.02, r = 0.2) (Fig 3D), CMV (p = 0.03, r = 0.2) (Fig 3F) and TMV (p<0.001, r = 0.3) (Fig 3H). Similar results were obtained on univariate regression analysis (Table 4). Insignificant correlation was found between OCT parameters and CVI, as indicated by the r-value close to zero (Fig 3C, 3E and 3F) and on multivariate regression analysis (Table 5).

An inverse correlation was found between SFCT and CVI, though statistically insignificant. (Fig 3I, **p<0.9**). Hypertensive patients were significantly found to have thinner SFCT when compared with non-hypertensive patients (319.0±54.6 μm vs 354.0±48.8 μm, p = 0.01). CVI was not affected by the presence of hypertension (64.1±1.8 vs 64.2±1.6, p = 0.71).

## Discussion

The choroid is responsible for blood supply to outer retinal layers. Disruption of its structure or vasculature can affect the retinal function. SFCT as measured on EDI OCT has been used as a surrogate marker for assessing the health of the choroid in patients with diabetes and other retinal disorders. Changes in diabetic choroidal vasculature have been described as increased tortuosity, dilatation and narrowing, hypercellularity, microaneurysm formation, drop-out of choriocapillaries, and sinus-like structure formation between choroidal lobules [15]. Studies have reported that choroidal blood flow decreases in patients with diabetes, especially with DME and more in PDR [25]. Previous published studies have reported varying results (thinning or thickening) in SFCT among diabetic patients. Kim et al [5] found that SFCT increases with severity of DR in the Korean population. Sudhalkar et al [6] found choroidal thinning with increasing severity of retinopathy in Indian population. Similar findings of a decrease in SFCT in patients with DR were seen in studies by Vujosevic et al [7], Laíns et al [8], Galgauskas et al [9] and Lee et al [10]. A large population study [11] in China reported choroidal thickening in patients with diabetes, but DR was not found to be associated with increased SFCT. A similar finding of no difference in SFCT between patients with DR and normal patients was seen in the American population studied by Regatieri et al [12]. A similar discrepancy in results was seen in patients with DME. Rewbury et al [26] showed non-statistically significant increase in SFCT in patients with DME, while Gerendas et al [27] showed a significantly reduced SFCT in patients with DME. The inconclusive nature of the results seen in the various studies shows that SFCT does not correlate well with the presence of DR or DME. We postulate that this poor correlation is due to SFCT being affected by many other systemic and ocular variables such as age, axial length, intraocular pressure and blood pressure [28].

In our study, we used CVI as the additional comparative tool to SFCT to understand the structural changes in the choroid in diabetic population. CVI is a novel tool, that represents the proportion of choroidal vasculature including both large choroidal vasculature and choriocapillaris [18–19]. Unlike SFCT, CVI is not shown to be affected by systemic and ocular factors mentioned above as demonstrated in our study [21].

Previous studies by Tan et al [20] and Kim et al [21], showed that there is a significant decrease in CVI in patients with DM and DR, but both the studies did not analyze any specific subgroup of DM patients with DME. In this current study, we showed that patients with DR and DME had a statistically significant decrease in CVI as compare to the healthy study group, which is similar to study by Kim et al [21]. We also analyzed the trend with increasing severity of DR and noted a decreasing CVI with increasing severity of DR. A similar trend was also observed by Kim et al in their study with varying grades of DR [21]. It has been shown by Cao et al [15] that there is 4 times more choriocapillary loss in diabetic eyes compared to non-diabetic eyes. A decrease in choriocapillary layer (that causes a decrease in CVI) may lead to hypoxia of the RPE and outer retina, leading to the upregulation of vascular endothelial growth factor [29], hence worsening of DR. Ferrara et al demonstrated a loss of intermediate and large blood vessels in the Sattler’s layer and Haller’s layers in diabetic eyes, seen previously with histological techniques [30]. The decrease in the intermediate and large blood vessels in patients with DM would result in a decrease in CVI, as seen in our study. Nagaoka et al also showed a decrease in choroidal blood volume and velocity in patients with DME [17]. It is also postulated that this decrease in choroidal flow, may lead to hypoxia of the retinal pigment epithelium and outer retina in patients with DME, leading to an increase in vascular endothelial growth factor, that induces the breakdown of blood retinal barrier, resulting in DME. Also, it is difficult to propose the correlation of choroidal thickness or CVI with retinal parameters and postulate a causal association. The increase in choroidal thickness might be the cause of retinal edema or the result of fluid accumulation because of the same underlying factors that drive the retinal edema also. The possibility of either is well reflected by the positive correlation between the SFCT and the measures of retinal edema (central macular thickness, central macular volume and total macular volume) in the present study.

However in the study by Kim et al, higher CVI was associated with lower central retinal thickness and higher SFCT [21]. As CVI is not affected by any of the above retinal parameters, it seems that there is a proportionate increase in both stromal and vascular area in the choroid, which is reflected in increased SFCT. Amongst the various types of DME, we observed that eyes with SRD had the thickest choroid. The current study findings are consistent with a previous study by Kim et al [5]. We also analyzed if SFCT and CVI were affected by the presence of hypertension. We found that hypertensive patients had thinner choroid as compared to non-hypertensive patients, in the diabetic group. However, CVI was not affected by hypertension. We postulate that CVI may be representative of the microangiopathic changes (vascular constriction and capillary dropout) seen in diabetes and DME while SFCT may represent the secondary changes of edema and leakage that may be seen because of hypoxia with a subsequent increase in VEGF. A significant increase in SFCT with the amount of DME as measured by a positive correlation with OCT parameters like CMT, CMV, TMV may also suggest that similar factors may be the driving force behind DME as well as increase in SFCT. CVI may be more indicative of the severity of underlying disease or diabetic microangiopathy, and may be unaffected by secondary changes due to VEGF release. Changes in the choroid are dynamic in eyes with DR and DME and may be important in the pathogenesis of retinal changes in DR and DME. With the improvement in the visualization of the choroidal morphology as a whole, using SD-OCT with EDI, analysis of CVI may be the first step towards understanding the pathogenesis of these changes in DME. The significant results seen from our study provides us with added information of choroidal changes in DME.

The strength of our study is inclusion of treatment naïve patients. However, the small sample size and absence of a comparative group of NPDR without DME are limitations of this study. A larger sample size will be needed to confirm our findings. Another limitation of our study is that correlation with physiological variables such as axial length and refractive error was not performed. Additional information may be derived if a volume scan over an area of the macula was obtained instead of an area scan over a linear section of the macula, the latter being used in our study. However, we have shown in our recent publication that there is statistically insignificant difference between the volume scans and the area scans [31]. Longitudinal analysis can further establish the value of CVI, however we could not perform longitudinal follow up of this study cohort. In our study, the eyes had thicker choroid and the results hence cannot be generalized to eyes with thinner choroid.

## Conclusion

Using CVI as a tool to analyze the choroid in patients with DME can potentially be useful in monitoring the progression of DME and DR in a non-invasive way, as an adjuvant to our current grading system based on clinical examination. CVI can also possibly provide us with greater insight to the pathogenesis and pathophysiology of the disease by indirectly tracking the changes in the choroidal vasculature as the disease occurs and progresses.
